# Beetles as Model Organisms in Physiological, Biomedical and Environmental Studies – A Review

**DOI:** 10.3389/fphys.2019.00319

**Published:** 2019-03-28

**Authors:** Zbigniew Adamski, Sabino A. Bufo, Szymon Chowański, Patrizia Falabella, Jan Lubawy, Paweł Marciniak, Joanna Pacholska-Bogalska, Rosanna Salvia, Laura Scrano, Małgorzata Słocińska, Marta Spochacz, Monika Szymczak, Arkadiusz Urbański, Karolina Walkowiak-Nowicka, Grzegorz Rosiński

**Affiliations:** ^1^Department of Animal Physiology and Development, Institute of Experimental Biology, Faculty of Biology, Adam Mickiewicz University in Poznań, Poznań, Poland; ^2^Laboratory of Electron and Confocal Microscopy, Faculty of Biology, Adam Mickiewicz University in Poznań, Poznań, Poland; ^3^Department of Sciences, University of Basilicata, Potenza, Italy; ^4^Department of Geography, Environmental Management & Energy Studies, University of Johannesburg, Johannesburg, South Africa; ^5^Department of European and Mediterranean Cultures, University of Basilicata, Matera, Italy

**Keywords:** beetles, model organisms, bioactive compounds, agronomy, immunology, neuroendocrinology, biomonitoring

## Abstract

Model organisms are often used in biological, medical and environmental research. Among insects, *Drosophila melanogaster, Galleria mellonella, Apis mellifera, Bombyx mori, Periplaneta americana*, and *Locusta migratoria* are often used. However, new model organisms still appear. In recent years, an increasing number of insect species has been suggested as model organisms in life sciences research due to their worldwide distribution and environmental significance, the possibility of extrapolating research studies to vertebrates and the relatively low cost of rearing. Beetles are the largest insect order, with their representative – *Tribolium castaneum* – being the first species with a completely sequenced genome, and seem to be emerging as new potential candidates for model organisms in various studies. Apart from *T. castaneum*, additional species representing various Coleoptera families, such as *Nicrophorus vespilloides, Leptinotarsa decemlineata, Coccinella septempunctata, Poecilus cupreus, Tenebrio molitor* and many others, have been used. They are increasingly often included in two major research aspects: biomedical and environmental studies. Biomedical studies focus mainly on unraveling mechanisms of basic life processes, such as feeding, neurotransmission or activity of the immune system, as well as on elucidating the mechanism of different diseases (neurodegenerative, cardiovascular, metabolic, or immunological) using beetles as models. Furthermore, pharmacological bioassays for testing novel biologically active substances in beetles have also been developed. It should be emphasized that beetles are a source of compounds with potential antimicrobial and anticancer activity. Environmental-based studies focus mainly on the development and testing of new potential pesticides of both chemical and natural origin. Additionally, beetles are used as food or for their valuable supplements. Different beetle families are also used as bioindicators. Another important research area using beetles as models is behavioral ecology studies, for instance, parental care. In this paper, we review the current knowledge regarding beetles as model organisms and their practical application in various fields of life science.

**GRAPHICAL ABSTRACT:**
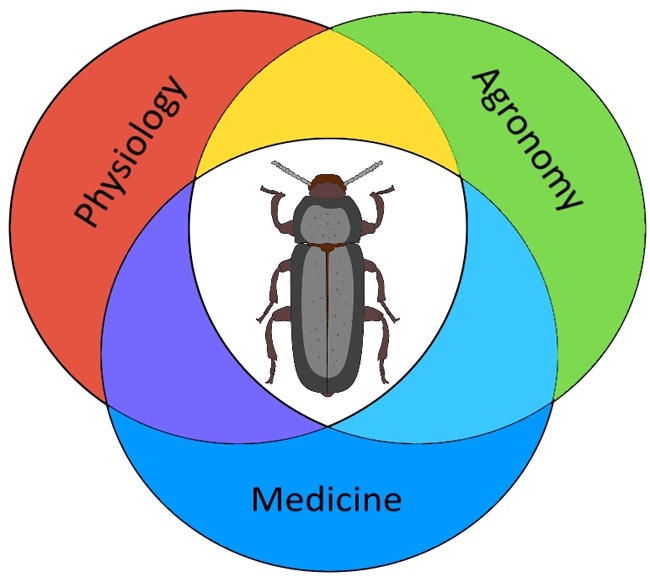
Beetles at the center of life sciences, more and more often used as model organisms.

## Introduction

Insects are novel model organisms to examine human diseases and to establish drug toxicity due to the conservation of their signaling pathways, energy metabolism and structural components with mammals. Furthermore, insects are cost-effective, easy to rear and time-efficient in the early steps of drug discovery and physiological processes investigation. For over 100 years, the fruit fly, *Drosophila melanogaster*, has been a powerful tool in genetic, behavioral and molecular biology studies. Beyond *Drosophila*, some other insects such as *Galleria mellonella, Bombyx mori, Periplaneta americana*, and *Locusta migratoria* have been used in biological, medical and environmental research (Stankiewicz et al., 2012; Wang et al., 2014; Soumya et al., 2017; Mikulak et al., 2018). In addition to these insect species, scientists have been recently searching for new model insects for studies in physiological and environmental studies.

During the last decade, the interest of scientists on the Coleoptera order increased, the richest order in the animal kingdom with approximately 400,000 species constituting almost 25% of all known animal life-forms and occupying most terrestrial environments (Stork et al., 2015). Beetles interact with plants and other organisms as well as dead and decaying biotic materials, thus playing a key role in natural and human-dominated ecosystems (McKenna et al., 2009). They have survived major floral and faunal disasters and several mass extinctions over their 300 million years of history (McKenna et al., 2016). Furthermore, many beetle species are serious pests in many areas of the world, leading to significant losses in the nutritional value of plants and agricultural products (Tribolium Genome Sequencing Consortium, 2008). Therefore, the sequencing of some beetle genomes has provided a powerful tool for revealing genomic innovations contributing to the evolutionary success of beetles and understanding of insecticide resistance mechanisms, the specificity and effectiveness of insecticides and biological agents, the interactions of host plants with microorganisms, and, finally, the development of new protection methods.

So far, 11 genomic sequences are available for 11 beetle species; among them, 7 have been published (McKenna, 2018). The first sequenced beetle genome was the red flour beetle, *T. castaneum*, an important pest of agricultural products (Tribolium Genome Sequencing Consortium, 2008). The *T. castaneum* genome contains large expansions of odorant receptors, gustatory receptors, and genes putatively involved in detoxification, which reflects the ability of the pest to interact with a diverse chemical environment. Some developmental processes of *T. castaneum* are more representative and comparable to those of mammals than those of *Drosophila* (Schroder et al., 2008). The ancestral genes involved in cell-to-cell communication expressed in the growth zone crucial for axial extension in short-term development make *T. castaneum* ideal for studying the evolution of development, such as segment specifications (Von Dassow et al., 2000) or multilevel selection (Goodnight, 2005). Genetic knowledge, and, in particular, easy genetic manipulations of gene expression RNA interference (RNAi) (Bucher et al., 2002; Knorr et al., 2018) at different stages of *T. castaneum* development make this beetle an even more valuable model in a number of research fields, for example, identification of targets for selective insect control. Currently, *T. castaneum* is a one of the most convenient genetic models for post-genomic studies such as RNA expression profiling, proteomics and functional genomics, mechanisms of insect immunity (Altincicek and Vilcinskas, 2007), gene–food interactions (Grunwald et al., 2013) or host–pathogen relationships (Milutinović et al., 2013).

A short time after the *T. castaneum* genome was sequenced, other beetle genomes were also sequenced; in 2013, the mountain pine beetle, *Dendroctonus ponderosae*, an important forest pest (Keeling et al., 2013), and in 2015, the coffee berry borer, *Hypothenemus hampei*, and the burying beetle, *N. vespilloides*, (Cunningham et al., 2015b; Vega et al., 2015) genomes were published. In their genomes, genes putatively involved in detoxification and plant cell wall degradation, as well as gene of bacterial sucrose-6-phosphate hydrolase, with proposed relevance to digestive physiology have been found. Furthermore, it was found that the *N. vespilloides* genome has an active DNA methylation system and genes responsible for social behavior. The genomes of *Oryctes borbonicus* (Meyer et al., 2016) and *Anoplophora glabripennis* (McKenna et al., 2016) were published in 2016.

Of particular interest is the sequencing of the genome of the Colorado potato beetle, *Leptinotarsa decemlineata*, one of the main pests that is difficult to manage, which has a special ability to adapt to solanaceous plants and changing environmental conditions (Schoville et al., 2018). The *L. decemlineata* genome analysis supports a rapid evolutionary change and confirms the genomic basis of phytophagy and insecticide resistance. It was found that *L. decemlineata* has evolved resistance to over 50 pesticides. Adaptations to plant nutrition have been observed in gene expansions that are putatively involved in digestion and detoxification, as well in gustatory receptors for bitter taste. The spectacular ability to exploit novel host plants and unusual resistance together with the newly released genome make *L. decemlineata* a good model system for agricultural pest genomics and developing sustainable methods to control this pest (Schoville et al., 2018).

Sequencing of genomes combined with the development of proteomic techniques such as mass spectrometry enables the understanding of the evolutionary and genomic basis of the biodiversity of beetles’ genomes and importantly, applying new technologies to the exploitation of beetle genomes for human purposes. However, the majority of beetles intensively developed in many research areas, e.g., *Tenebrio molitor, Coccinella septempunctata, Poecilus cupreus* and many others, still have unknown genomes. We suppose that it is only a matter of time because beetles are convenient and inexpensive animal models, invaluable for studying physiological processes during insect development and biomedical and pharmacological research between invertebrates and vertebrates.

Many physiological mechanisms of insects exhibit a high level of similarity or identity with higher animals (Altincicek et al., 2008; Ntwasa et al., 2012). The neuroendocrine system exhibits similarities at the structural, functional and developmental levels between beetles and vertebrates (Hartenstein, 2006). Signaling molecules such as hormones and neuropeptides of insects have physiological counterparts in mammals, indicating that they are highly conserved in the animal kingdom. This fact provides an opportunity to perform comparative studies between invertebrates and vertebrates concerning neurobiology and the role of signaling molecules in animal physiology. This approach may be promising for the treatment of many human diseases, such as obesity, metabolic syndrome or cardiovascular diseases (Chowański et al., 2017a).

Furthermore, beetle models have been developed at the early steps of pharmacological studies for testing the activity of new substances of both synthetic and natural plant or animal origin. Moreover, Coleopterans themselves are a source of compounds with a potential antimicrobial and antitumor activity, which could likely be used in cancer therapy and immunology (Słocińska et al., 2008; Chowański et al., 2017a; Walkowiak-Nowicka et al., 2018). For these reasons, the use of beetle model systems for screening of drugs and active agents seems to be reasonable.

Vertebrate models have many limitations: long generation time, low fecundity, high housing costs and ethical permissions. Recently, *T. castaneum* has been used as an early warning system for environmental effectors, such as nutrients or pharmaceutics, effects on the health and lifespan of living organisms (Denell, 2008; Grunwald et al., 2013; Bingsohn et al., 2016). Additionally, beetles are used as food or sources of valuable food supplements.

In this paper, we review the current state of knowledge regarding beetles as model organisms in biomedical and environmental studies. We show the applications of beetle models in different fields of research, such as neuroendocrinology, immunology, pharmacology and toxicology. Moreover, we indicate the importance of beetles in environmental studies including agriculture, food production or ecosystem monitoring, which are closely interrelated with physiological studies as well.

## Neuroendocrinology

**FIGURE 1:**
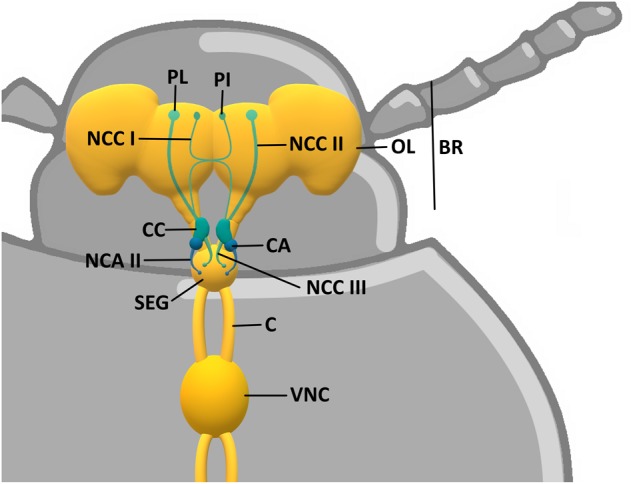
Scheme of *Tenebrio molitor* neuroendocrine system. BR, brain; OP, optical lobe; PI, *pars intercerebralis*; PL, *pars lateralis*; NCC I, NCC II, and NCC III, *nervi corporis cardiaci* I, II, and III; CC, *corpora cardiaca*; CA, *corpora allata*; NCA II, *nervi corporis allati* II; SEG, suboesophageal ganglion; C, connectives; VNC, ventral nerve cord.

Through the process called neuroendocrine integration, the nervous system and the endocrine system interplay together in regulation of function and homeostasis maintenance. Different studies have emphasized the similarities between the neuroendocrine system of vertebrates and arthropods (especially insects, including beetles) at the structural, functional, and developmental levels (Hartenstein, 2006; De Loof et al., 2012). The hormone-producing neurons are called neurosecretory cells (NSCs). In vertebrates, they are located mainly in the hypothalamus. NSCs, besides innervating brain centers and thereby influencing neural circuits as “neuromodulators,” send their axons to the peripheral neurohaemal glands (posterior pituitary gland) in which hormones produced by the NSCs are stored and released. The stimulatory neurohormones are also transported by the portal blood vessel system to the anterior pituitary, where they cause the release of a specific hypophyseal hormone (McKinley et al., 2016). Insects do not have any region of a brain that can be considered as a morphological and physiological counterpart of the hypothalamus, nor any portal blood vessel system in their brain (De Loof et al., 2012). However, the corresponding structures would be groups of NSCs located in the brain protocerebrum, such as the *pars intercerebralis* (PI), *pars lateralis* (PL), or tritocerebrum. NSCs are also located in the suboesophageal and/or ventral nerve cord ganglia. PI/PL project their axons toward peripheral targets, which are the paired neurohaemal organs *corpora cardiaca* (CC) and *corpora allata* (CA) situated in the beetle’s head capsule on both sides of the proximal end of the aorta, and form a retrocerebral complex (Sliwowska et al., 2001; Urbański et al., 2018). Like the mammalian pituitary gland, the CC of insects/beetles consists of two distinct lobes: an unpaired ventral storage lobe, containing the NSCs’ terminals located in the PI and PL, and a more lateral glandular neurohaemal lobe, which also has its own NSCs (Figure 1).

Despite anatomical and functional similarities in the neuroendocrine system, there is a great resemblance of the signaling molecules of invertebrates and vertebrates, which is summarized in: (Chowański et al., 2017a). Most of the hormones or neurohormones found in the animal kingdom are short polypeptides produced by proteolytic cleavage from larger precursor proteins, called prohormones. Major examples of neuropeptides found in insects/beetles and their analogs in vertebrates are adipokinetic hormones (AKHs) that resemble mammalian glucagon, insulin-like peptides (ILPs) that resemble mammalian insulin, tachykinin-related peptides (TRPs) that resemble mammalian tachykinins, and many others (Chowański et al., 2017a). Considering all the above described structural and functional similarities, an increasing number of studies has been conducted under the neuroendocrine regulation of various physiological processes. There are two main areas of interest in biomedical aspects: (a) the use of simple insect models of the neuroendocrine system for detailed study of the complex mechanisms of neurohaemal regulation in mammals and other higher vertebrates to reveal the principles of various disorders; (b) a source of biologically active substances as a basis for new agents used in therapy of disorders. Below, we describe some examples of research conducted on insect neuropeptides in the context of biomedical studies.

### Food Intake

Feeding behavior and energy homeostasis is under strict neuroendocrine and endocrine control (Monteleone et al., 2018). Key players in this process in vertebrates are the so-called metabolic hormones, such as leptin, ghrelin or cholecystokinin (CCK), as well as neuropeptides, e.g., neuropeptide Y or orexins (Monteleone et al., 2018). In invertebrates, there are several analogs and homologs of these molecules that are similar on both the structural and functional level and sometimes common in origin. Sulfakinins (SKs) are insect homologs of mammalian gastrin/cholecystokinin (Rehfeld, 2017). They have been identified in various insects in two forms: containing sulfated or non-sulfated tyrosine (Nässel and Williams, 2014). This structural similarity together with the well-conserved *C*-terminal amino acid sequence HMRFa puts SKs in the family of CCK-like peptides. In mammals, CCK is a pleiotropic peptide responsible for various physiological processes, mainly as a regulator of satiety signaling (Rehfeld, 2017). Similarities between CCK and SKs were also found at the functional level. Studies on the beetles *T. castaneum* and *Zophobas atratus* showed that SKs inhibit food consumption and regulate energy metabolism in these species and hence may be a part of the insect brain-gut axis (Yu et al., 2013; Słocińska et al., 2015b). CCK signaling disorders in humans are associated with various illnesses, such as obesity or depression (Coulter et al., 2017; Klockars et al., 2018). Thus, SKs may be a good source of potent agents used for the treatment of these diseases in the future. This approach is even more possible since a similarity between CCK receptors and SK receptors has been shown (Słocińska et al., 2015a; Yu et al., 2015).

### Cardiovascular Diseases – A New Approach Using the Insect Heart

Cardiovascular disorders (CVDs) are among the most common diseases in the world. Therefore, it is important to gain extensive knowledge about the mechanisms that cause certain diseases or, on the other hand, find various substances that modify the activity of the heart to create a solid basis for further progress in the treatment of CVDs. Research on various insect models, including beetles, are very promising in this field (Szymczak et al., 2014; Zhu et al., 2017; Pacholska-Bogalska et al., 2018). The heart/myocardium of insects has been shown to be a convenient biomedical model. The spectrum of neurotransmitters, neuropeptides and peptide hormones that affect contractility and regulate normal heart rhythm is quite large (Chowański et al., 2016). Among them, many exhibit structural similarities to mammalian peptides/hormones (Chowański et al., 2017a). For example, myosuppressins, due to the highly conserved *C*-terminal amino acid sequence FLRFa, belong to the superfamily of RFamide peptides (Fukusumi et al., 2006). It has been demonstrated that mammalian RFamides inhibit the contractility of the vertebrate/insect myocardium (Nichols et al., 2012). The cardioinhibitory effects of myosuppressin were also observed in studies on the *Z. atratus* myocardium (Marciniak et al., 2010b). Small, highly specific molecules are the preferred candidates for drug discovery, partly due to delivery, synthesis and cost. In addition, the components of transduction mechanisms are targets for the development of therapeutic strategies for the treatment of diseases, such as heart failure. Furthermore, the insect G protein-coupled receptor for myosuppressin resembles mammalian GPCRs (Leander et al., 2015).

### Growth, Development and Reproduction Regulators

In insects, two groups of neuropeptides are proven to be involved in the regulation of growth, metamorphosis and reproduction by regulation of production and secretion of the juvenile hormone (JH) by CA (Feyereisen, 1985), stimulatory allatotropins or inhibitory allatostatins. Among allatostatins, three separate families can be distinguished, which vary structurally (Lubawy et al., 2018). The most interesting of these families for biomedical research seems to be the so-called PISCF/ASTC allatostatins. Structural similarities between mature somatostatin and mature PISCF/ASTCs are not very relevant. However, the resemblance between the vertebrate somatostatin/cortistatin precursors and the arthropod ASTC/ASTCC precursors consists of a disulfide bridge at the *C*-terminal end of these two precursors. Moreover, insect ASTC receptors are closely related to somatostatin receptors (Veenstra, 2009). In *T. castaneum*, ASTC receptor was shown to be shorter than other insect receptors but similar in size to human SSTR3 receptor (Audsley et al., 2013). ASTCs are thus considered arthropod somatostatin homologs (Veenstra, 2009). Next, the physiological resemblance is also evident. Human somatostatin acts on different tissues in the human body and in general, is known as an inhibitory peptide (Gahete et al., 2010). The same situation occurs in insects, as was shown in beetle models. PISCF/ASTCs were shown to be inhibitory in *T. castaneum* and *T. molitor* (Abdel-latief and Hoffmann, 2010, 2014; Lubawy et al., 2013). These similarities provide a starting point for fundamental studies about the pharmacology of human somatostatin receptors (SSTRs). Insect allatostatin C sequences may yield useful peptides to produce new ligands. Ligands for SSTRs are still needed to treat different endocrinopathies such as Cushing’s disease or acromegaly (Langlois et al., 2018; Paragliola and Salvatori, 2018).

### Diuresis Regulators

Other neuropeptides that are of interest in biomedical research are nonapeptides, oxytocin, and arginine vasopressin. In mammals, they are produced and released by the posterior lobe of the pituitary gland and influence many tissues and organs. To date, it is known that oxytocin and vasopressin mediate a range of physiological functions that are important for osmoregulation, reproduction, complex social behaviors, memory and learning. Comparing amino acid sequences of mature peptides across invertebrates and mammals, it is clear that some positions are highly conserved. For example, they have the same length and position of Cys residues crucial for forming disulfide bonds (Gruber, 2014). Moreover, invertebrate receptors for vasopressin show a high degree of similarity to mammalian ones (Liutkeviciute et al., 2016). Recently, 260 species of arthropods were searched for the presence of the inotocin peptide GPCR signaling system, and it was shown that not all of the insect orders possess this signaling system. Trichoptera, Lepidoptera, Siphonaptera, Mecoptera, and Diptera lack the presence of inotocin genes, which suggests that this peptide-receptor system was probably lost in their common ancestors (Liutkeviciute et al., 2016). For this reason, beetles seem to be better models to study inotocin signaling than the insect commonly used in biomedical research, *D. melanogaster*. Among Coleoptera, all 20 analyzed species exhibited evidence for the presence of the inotocin signaling system (Liutkeviciute et al., 2016). Vasopressin-like peptides and their receptors were already characterized from the *T. castaneum* beetle and were shown to be crucial for diuresis regulation in this species (Aikins et al., 2008).

Owing to this structural and physiological importance, ligands of oxytocin and vasopressin receptors have potential therapeutic applications for novel treatment approaches to different osmoregulatory disorders such as diabetes insipidus, as well as cardiovascular or even mental disorders (Gruber, 2014; Di Giglio et al., 2017).

## Immunology

The immune system of insects is based primarily on innate immune mechanisms that might be divided into cellular and humoral responses. The cellular response includes all processes in which morphotic components of insect haemolymph, haemocytes, participate. We indicate three main cellular mechanisms: phagocytosis, nodulation and encapsulation (Strand, 2008; Urbański et al., 2014). The humoral response includes processes in which molecules such as antimicrobial peptides (AMPs), lysozyme or phenoloxidase (PO) participate, for which the concentration increases dramatically after a pathogen infection (Bulet et al., 1999; Wojda et al., 2009). However, distinction between these two types of immune response is artificial, because pathogen recognition by haemocytes is crucial for the activation of humoral responses, and humoral mechanisms stimulate the activity of haemocytes (Strand, 2008; Urbański et al., 2017).

**FIGURE 2:**
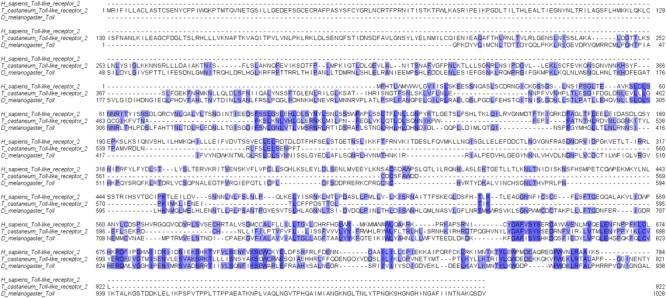
An exemplary comparison of similarity between important parts of insect and mammalian immune systems. Alignment of human Toll-like receptor 2 sequence and its structural homologs identified in *Tribolium castaneum* and *Drosophila melanogaster.* Color indicates identity in the amino acid sequence. The sequences of receptors were obtained from a BLAST search in a public database (NCBI) using the blastp algorithm to analyze the transcriptomic data of *Homo sapiens, T. castaneum* (AN: PRJNA15718) and *D. melanogaster* (AN: PRJNA164). The alignment was made using JalView software (ver. 2.10.).

The use of beetles as model organisms in biomedical studies is associated with the similarity of basic immune mechanisms, such as pathogen recognition, immunodeficiency, and molecular pathways such as Toll and JAK/STAT signaling (Altincicek et al., 2008; Vogel et al., 2014; Figure 2). In each of these mechanisms, their individual components have vertebrate counterparts, starting from the receptors (e.g., beetle Toll receptor and mammalian Toll-like receptor) and ending with transcription factors (e.g., transcription factor Dif characteristic for immunodeficiency pathways belongs to the NF-κB transcription factor family) (Maxton-Küchenmeister et al., 1999; Chen et al., 2000; Yokoi et al., 2012; Johnston et al., 2014). Moreover, recent studies generally concerning molecular similarities between insects and vertebrates indicated that beetles have retained many ancestral genes compared to those of other insect orders (Savard et al., 2006; Van der Zee et al., 2008). Many of these genes were found in vertebrates and beetles but not in fruit flies. Also, beetle immune-related genes often have a higher level of similarity and identity with vertebrate analogs than those observed in *Drosophila* species, for example, insect Toll protein and vertebrate Toll-like receptor. Furthermore, the similarities of the insect and vertebrate immune systems are present also in the regulation of their function, for example, by serpins (serine protease inhibitors) (Gooptu and Lomas, 2009; Jiang et al., 2011). All of these studies clearly indicate that beetles are useful models for studies concerning the molecular basis of the innate immune response and its evolution (Savard et al., 2006; Ntwasa et al., 2012). Moreover, the availability of the full genome sequence of several beetle species allows for genetic manipulation, including gene knockdowns using RNAi, and leads to unquestionable progress in the use of beetles in these kinds of studies (Rajamuthiah et al., 2015).

Similarities between beetles and vertebrates are also evident in the regulation of haemocyte activity during the cellular response. The similarity can be exemplified by the regulation of phagocytosis in both animal groups by focal adhesion kinase (FAK) and mitogen-activated protein kinase pathways (MAPK) (McLeish et al., 1998; Tang et al., 2014).

## Agronomy and Ecology

### Crop Growth, Food Production and New Sources of Nutrition

Beetles play an important role in agriculture and food production. However, their influence on these fields of studies can be positive and negative. In our review, we focus on the use of beetles as alternative sources of food and their role in maintaining a good condition of cultivated fields as well as their importance for ecosystem stability.

It is obvious that the present-day world needs to develop alternative sources of proteins for livestock feed as well as for the human diet, considering the production of high-protein foods is extremely expensive, due to the costs concerning money spent directly on animal farming and costs resulting from space-, water- and environment-consumption. The production of 1 kg of beef consumes 15,000 L of water and 330 m^2^ of soil and introduces 16.4 kg of CO_2_ into the environment (FAO, 2013). Moreover, livestock production is the world’s largest user of land (Foley et al., 2011). Thus, searching for new, less expensive sources of nutrient-rich and high-caloric food is a large challenge for contemporary society. Insects are of a great interest as a possible solution due to their capability to satisfy two different requirements: (1) they are an important source of protein and other nutrients and (2) their use as food has ecological advantages over conventional meat and, in the long run, leads to economic benefits (Belluco et al., 2013). Globally, the most commonly consumed insects are beetles (Coleoptera); they account 31% percent of insects used as a food. Overall, insect breeding should not compete for space or other resources with plant culture; instead that, it should complement it, e.g., insects may feed on plants or plant parts that are unsuitable for human consumption or on organic wastes (FAO, 2013). It is also suggested that insects can be a huge source of plant cell wall degrading enzymes (Pauchet et al., 2010; Busch et al., 2017). Therefore, insects are able to contribute significantly to cycles of matter (Katayama et al., 2008). In this way, organic wastes can be used for feed production *via* insects. Ramos-Elorduy et al. (2002) used *T. molitor* larvae reared on organic wastes as a supplementation to the diet of broiler chickens. Dried and milled larvae were added to fodder at a concentration of 5 or 10% of total weight. The addition of larvae to the basic diet did not significantly affect food intake, weight gain, or feed efficiency (Ramos-Elorduy et al., 2002). Moreover, addition of fermented *T. molitor* and *Z. atratus* to broiler meal lead to reduction of caecal *Escherichia coli* and *Salmonella spp*. and increased IgG and IgA levels. Most likely, these effects are combined results of chitin and probiotics contained in this forage (Islam and Yang, 2016; Gasco et al., 2018). Similar results were also obtained in studies on European sea bass fingerlings, in which *T. molitor* enhances lysozyme activity (Gasco et al., 2018; Henry et al., 2018). Beetle forage may also be used as meal for aquatic invertebrates. Panini et al. (2017a,b) showed that replacing fishmeal with mealworm meal does not alter protein content in the muscles of *Litopenaeus vannamei* shrimp, but it increased the lipid content. However, for a human nutritional perspective, the lipid content of the shrimps is considered low and within the optimal concentration (Panini et al., 2017a,b). An advantage of insects as a food/fodder is that they have higher feed conversion efficiency in comparison to conventional livestock, i.e., they need less feed to produce 1 kg of biomass (Rumpold and Schluter, 2013). For example, the average daily gain of *T. molitor* larvae is 2 and 25 times higher than that for pigs and beef cattle, respectively, and they produce much less CO_2_ (g/kg mass gain) than the mentioned livestock. Moreover, the production of other greenhouse gasses (N_2_O, CH_4_, and NH_3_) is also significantly lower (Oonincx et al., 2010). Life cycle assessment studies covering all energy costs of biomass production by organisms have been performed (e.g., food consumption, feed production, transport, electricity, etc.). Studies conducted by Oonincx and de Boer (2012) showed that production of 1 kg of dry biomass and production of 1 kg of edible proteins by larvae of two beetle species, *T. molitor* and *Z. atratus*, requires definitely fewer natural resources than cattle, pigs and chicken. Thus, for every 1 ha of land required for producing mealworm protein, 2.5 ha would be required for producing a similar amount of milk protein, 2–3.5 ha for pork or chicken protein, and 10 ha for beef protein (FAO, 2013). The efficiency of food conversion into biomass may significantly differ in insects depending on the diet composition. However, insects can be bred on organic byproducts, wastes which cannot be used in the case of pigs, sheep, or beef cattle. Larvae of three tenebrionid beetles, *T. molitor, Z. atratus*, and *Alphitobius diaperinus*, fed with organic byproducts from beer brewing, bread/cookie baking, potato processing and bioethanol production still retained high efficiency of food conversion into biomass comparable to that of conventional livestock (van Broekhoven et al., 2015).

Beetles exert an important impact on the condition of cultivated fields. They take part in pest management and soil fertilization and reduce weeds. Two groups are especially important: dung beetles (Scarabaeoidea) and ground beetles (Carabidae). Scarabaeoidea, the coprophagous beetles that feed on animal excreta as both adults and larvae, provide several key functions to agroecosystems. By manipulating feces, dung beetles play an important role in the nutrient cycle, trophic regulation, soil fertilization and biological pest control (Nichols et al., 2008). Studies on the dung beetle, *Onthophagus lenzii*, showed that the beetles’ activity significantly affects cow dung decomposition. In the first 5 days, rapid organic nitrogen decomposition is noted, regardless of dung beetle activity. After 15 days, dung beetles accelerate or initiate another ammonification process in residual cow dung and dung balls. They do so by producing aerobic conditions through a rapid decrease in moisture content (Kazuhira et al., 1991). Thus, a soil is fertilized with nitrogen, sulfur and phosphorus forms available for plants. Bang et al. (2005) found that adding one of three tested dung beetle species (*Copris ochus, C. tripartitus*, and *O. lenzii*) to cultivated fields increased the annual yield of perennial ryegrass by approximately 20%, and adding all of the species together gave 11% greater yield than that of the control.

The second mentioned group, the ground beetles, has an effective role in weed reduction (granivorous and omnivorous) and pest control (predators). Among all agricultural practices, the management of weeds has historically been the most resource-demanding practice performed by growers (Kulkarni et al., 2015). Seed consumption by Carabidae beetles (e.g., *Pseudoophonus rufipes, Harpalus affinis*, and *Amara aenea*) can help to reduce seed stock of a weed species in the range of 65 to 90% (Honek et al., 2005). Weed seed consumption rates of up to 74% have been documented for *Viola arvensis, Stellaria media*, and *Capsella bursa-pastoris* in agricultural habitats (Jonason et al., 2013). Within plant-feeding carabids, two “functions” can be distinguished: (1) utilization of green plant parts and fruits for supplementing beetles and (2) feeding on seeds by so-called “spermophagus” species (Kulkarni et al., 2015). The second group of Carabidae beetles forms a large group of formidable predators in the insect world. The following species might be given as examples: *P. cupreus, Bembidion lampros, Trechus quadristriatus, Carabus auratus*, and *Pterostichus melanarius*. Carabidae beetles living on the surface of the soil capture and consume a wide assortment of soil dwelling insects. The potential of carabids for pest control has been recognized a long time ago by observant farmers (Holland, 2002). Exemplary data show their importance. Field data indicate that Carabidae beetles’ predation caused an 81% decrease in emerging adults of *Sitodiplosis mosellana* and their parasites (Kromp, 1999). The field population of eggs and first-instar larvae of the cabbage root fly (*Delia radicum*) might even be reduced by 90% by such carabids as *B. lampros* and *T. quadristriatus* (Finch and Elliott, 1992). Another result shows that the population of *L. decemlineata*, in presence of such Carabidae species as *C. auratus* or *P. melanarius*, undergoes reduction of up to 30% (Kromp, 1999).

### Pest Management

Among pests, beetles are the one of the most important and largest groups, often with global importance (Spochacz et al., 2018). Therefore, many studies are carried out to check the effect of pesticides against them. Development of resistance to pesticides focuses interest of scientists, from the fields of life sciences to economy and law. *L. decemlineata* has developed resistance against 56 insecticides, *T. castaneum* against 32, and an important pest of rape, *Meligethes aeneus*, against 27 ones (Arthropod-Pesticide-Resistance-Database, 2018). Due to their resistance to numerous xenobiotics, the beetles are models for testing various aspects of this phenomenon, including genetic, enzymatic, physiological and behavioral (Zhang et al., 2012; Crossley et al., 2017; Schoville et al., 2018), as well as models of the evolutionary processes to explain the basis of development of resistance (Pelissie et al., 2018). The level of resistance may increase as much as 2,000 times (Alyokhin et al., 2008). The phenomenon has been studied on economically important beetles: *L. decemlineata, Eriopis connexa, Cryptolaemus montrouzieri, M. aeneus*, and *Sitophilus zeamais* (Guedes et al., 2006; Alyokhin et al., 2008; Zhang et al., 2012; Rodrigues et al., 2013; Stratonovitch et al., 2014). The mechanisms of resistance include alterations at various levels of biological organization and lead to altered behavior and reorganization of physiological processes or structure of cuticle. Therefore, detoxification requires an investment of large amounts of energy. The trade-off of energy between detoxification and insect development is an important topic in the research on resistance. Guedes et al. (2006) suggested that insecticide-resistant beetles are characterized by significantly higher mobilization of energy reserves. The different energy management of the resistant and susceptible strains manifests also, for example, in a different structure of trophocytes. This finding suggests that the metabolism of resistant strain is biased toward detoxification rather than development. Therefore, insecticides and other xenobiotics slow down insects’ development, inhibit molting, and decrease emergence (Weissenberg et al., 1998; Adamski et al., 2005; Chaieb, 2010; Barbosa et al., 2018). Some tests indicated that resistance to insecticides may lead to decreased fitness in the insecticide-free environment and results in decreased generation-time and differentially biased sex ratio (Gordon et al., 2015).

Ultrastructural and enzymatic malfunctions and malformations are correlated with the toxicity of insecticidal compounds, including natural ones. Beetles are used also as model organisms to help explain their mode of action. The involvement of esterases (Cutler et al., 2005) and factors which regulate cytochromes (Kalsi and Palli, 2017) in detoxification of pesticides was proved using beetles. The activity of several enzymes important in pesticide resistance, such as glutathione *S*-transferase, superoxide dismutase, and catalase, was described in model beetle organisms (Papadopoulos et al., 1999; Kostaropoulos et al., 2001; Adamski et al., 2003; Kolawole and Kolawole, 2014; Lambkin and Furlong, 2014; Abdelsalam et al., 2016). Several ultrastructural effects in non-target tissues were described in coleopterans: condensation of chromatin, disruption of biological membranes or malformations of mitochondria (Adamski et al., 2005; Abdelsalam et al., 2016). Hence, cyto-physiological alterations are key reactions explaining the mechanisms of toxicity but also the development of resistance in pests.

Cutler et al. (2005) indicate that pests may be resistant to pesticides that have not been widely used yet. For example, cross-resistance was observed for *L. decemlineata* in Canada (Scott et al., 2015). This phenomenon is of a huge importance to crop protection. Therefore, beetles have also been taken into consideration during construction of mathematical and computer models that simulate the development of resistance and to plan strategies of pesticide management (Argentine et al., 1994; Clift et al., 1997; Stratonovitch et al., 2014; Sudo et al., 2018). *L. decemlineata* has been proposed as an important species for the development of predictive strategies to minimize the resistance to pesticides (Kitaev et al., 2017). This species is also a reference model in research, where resistance of two or more species is confronted (Arain et al., 2017; Spit et al., 2017).

Beetles are amongst the most important species, both as pests and as predators, that limit herbivory. Therefore, regulation of their reproduction, fertility, fecundity, and development is a focus of interest in plant protection and agronomy and is crucial for integrated pest management programs. Mating behavior includes items such as partner recognition, courtship, and copulation. One of the most studied groups is Coccinellidae because of its important role in the reduction of aphids. Lately, the invasiveness of *Harmonia axyridis* has been observed. The sexual behavior of this species has been described as a 5-step process: “approach,” “watch,” “examine,” “mount,” and “attempt to copulate” (Obata, 1987). In another ladybird species, *Adelia bipunctata*, no “watching” step occurs (Hemptinne et al., 1998). A peculiar sexual behavior was observed in *Tenuisvalvae notata*, where a male spins two times on the back of a female after the copulation, which was not connected with body adjustment (dos Santos et al., 2017). Pheromones, vision and acoustic signals for communication between two individuals play a very important role in sexual behavior. The sexual behavior of lady beetles relies on pheromones; during the female reproductive cycle particular compounds can be identified. These compounds can be used to manipulate the behavior of insects, and due to this effect, they can be applied as new insect pest management strategies (Fassotte et al., 2016). Some insects, for example, bark beetles, use courtship songs during mating. According to Lindeman and Yack (2015), male *Dendroctonus valens* (Curculionidae) produce acoustic signals while approaching the female gallery. In this way, a female can choose a male grounding on the quality of the male song. The abovementioned studies may inform the use of non-chemical attractants in the future.

An interesting application of beetles in pest management was described for ambrosia beetles, a group of the subfamilies of Scolytidae and Platypodinae (Curculionidae) that are known for cultivating fungi in the galleries they dig inside tree barks. Most of the species of ambrosia beetles attack damaged or dead trees without any negative economic effect. However, there are species (*Platypus koryoensis, Xylosandrus compactus*, and *Austroplatypus incompertus*) that are invasive to new habitats and may cause significant losses (Vannini et al., 2017). *A. incompertus* is able to infest healthy, undamaged trees, decreasing the quality of wood. After infestation, the cultivation process is maintained with symbiotic fungi whose spores are released by insects from special morphological structures, the mycangia. In this way, ambrosia beetles are also vectors of fungal infections among trees (Moon et al., 2012). Recent studies have shown that ethanol present in trees promotes the growth of fungi and inhibits the appearance of other non-symbiotic fungi such as *Aspergillus*. Hence, ambrosia beetles choose trees with stem tissues containing ethanol to increase the effectiveness of food production (Ranger et al., 2018). This compound also attracts another ambrosia beetle, *Trypodendron lineatum*, and therefore, it can be used for pest management (Moeck, 1970).

*Harmonia axyridis* is known to be an invasive species in many countries that contains the native species *A. bipunctata* and *C. septempunctata*. The latest research showed the existence of factors that can promote the invasiveness of harlequin lady beetles (Verheggen et al., 2017). One of them is aggregation behavior, which appears among adults and pupae *H. axyridis*. It has been proved that gregarious pupation decreases mortality in pupae (Roberge et al., 2016) and that adult beetles can aggregate both in wintering and non-wintering conditions (Durieux et al., 2015). Additionally, it was documented that individuals of this species follow objects with highly contrasting colors (Nalepa et al., 2005).

Altogether, the complex, multilevel investigations on insect physiology and behavior may lead to the discovery of novel substances and manners for pest management. In addition, in the field of pesticides and their effects on beetles, in-depth studies focusing on both main and side effects, from the biochemical level through cellular, physiological and organismal effects and up to the ecosystems, are necessary for the proper estimation of their toxicity. The wide range of these studies may show the most realistic effects of pesticides on beneficial organisms.

### Biomonitoring

Heavy metals, pesticides, and even radiation alter most of the earth’s ecosystems. Therefore, many living organisms are used as models and bioindicators in the monitoring and assessing of the state of the environment and changes in ecosystems and provide warning signals for imminent ecological changes (Nouchi, 2012; Testi et al., 2012; Kalkan and Altuǧ, 2015; Parmar et al., 2016; Siddig et al., 2016). Among species that raise great interest in the field of ecosystem monitoring, beetles have started to attract attention as biological indicators of environmental contaminants (Zödl and Wittmann, 2003). The coleopterans may be found in almost every type of habitat in which any insect is found, and the niches that are inhabited by many species of Coleoptera are often inaccessible for other insects. Some beetle species are also immensely sensitive to changes occurring in their environments (Osman and Shonouda, 2017; Shonouda and Osman, 2018). Taking this fact into consideration along with well-established and standardized collecting methods (Spence and Niemelä, 1994) and readily available keys for identification, coleopterans are perfect organisms for bioindication. Ground beetles (Carabidae) are the most commonly used bioindicators, notably when trying to assess environmental pollution (Abildsnes and Tømmerås, 2000; Ghannem et al., 2018). Other families, such as Curculionidae, Tenebrionidae, and Staphylinidae, are also used (Shonouda and Osman, 2018). Agricultural activities strongly influence the assemblage of ground beetles found in woody habitats and grasslands. In general, more disturbed habitats support ground beetle fauna of smaller average body size (Petit and Usher, 1998). It has been shown that increased agricultural management leads to a decreased number of Carabidae forest species as well as number of individuals in hedgerows (Burel et al., 2004). Changes were correlated to the type of crops cultured between hedgerows. The relative abundance of large carabid species decreased, while small, mobile and more ubiquitous species were favored where cereal crops were cultured. However, some scientists report that even though total richness and composition of the species pools are the same, the rate of beetle species accumulation depends on the type of agricultural practice (Fahrig et al., 2011). Other researchers also showed that human influence, for example, cultivation of orchards, causes qualitative changes in beetle species composition in comparison to that of natural forest habitats (Allegro and Sciaky, 2003). Agricultural and veterinary practice can cause a change in beetle population, as was evaluated in Canada in a study considering endectocides (e.g., doramectin and eprinomectin; macrocyclic lactones that are veterinary parasiticides used to control nematodes and arthropods affecting livestock) (Floate, 2006). Cattle that were exposed to those products excreted metabolites, that were subsequently tested on dung beetles such as *Onthophagus nuchicornis* or *Aphodius prodromus*. The abovementioned endectocides, globally used in treating ectoparasites, reduced useful insect diversity and might cause the accumulation of dung on pastures (Floate, 2006).

Carabidae beetles can also be used as biodiversity indicators. The richness in beetle species can reflect the diversity of other taxa inhabiting a given environment. For example, Pearson and Cassola (1992) have shown the correlation between the richness of tiger beetles (Carabidae: Cicindelinae) and birds and butterflies. Carabid species richness has also been shown to have a positive correlation with that of other beetle families (Scarabaeidae and Pselaphidae) (Oliver and Beattie, 1996). Moreover, observations and quality-checking of beetle species richness and populations’ homogeneity/heterogeneity are also used to control the stability of unique habitats, for example, rainforests. Davis (2000) showed that usage of reduced-impact logging in the Malaysian rainforest leaves more equitable and species-rich communities of dung beetles. The dung beetle species richness is also notably higher in primary rainforests, where the majority of species are more abundant than in secondary ones, and plantation sites harbored an impoverished subset of primary forest species (Gardner et al., 2008). Knowledge about these correlations can be a useful tool in monitoring human influence on the natural environment.

Beetles can also be used for determination of heavy metal pollution. Elevated concentrations of lead and mercury were observed in zoophagous species and in some mycetophagous species of Staphylinidae, respectively (Bohac, 1989). Lagisz and Laskowski (2008) showed that an increase in zinc and lead levels in *P. oblongopunctatus* can cause a reduction in elytra length. This evidence shows that pollutants may affect body size, causing its decrease. This effect is probably the result of a negative effect on species development. Similar results were obtained for *P. cupreus* whose body size decreases notably with an increase of heavy metals in food (Maryanski et al., 2002). In addition to direct toxic effects, heavy metal pollutants may alter locomotory behavior, as was shown in larvae of *P. cupreus* exposed to copper (Bayley et al., 1995), and it affects reproducibility by causing degeneration of the flagella of spermatids (Shonouda and Osman, 2018).

Although coleopteran insects include the most important pests, many beetles are beneficial for ecosystems, and they are not regarded as destructive animals. Therefore, environmental studies often focus on the effects on whole beetle communities, observing alterations in the presence of beetle species and their behavior (Lee et al., 2001; Sivčev et al., 2017). Carabid beetles belong to the group of important soil-inhabiting species that are beneficial for soil quality. They prey on other insects; therefore they limit populations of herbivores. The species are noted, among others, in potato fields. Therefore, these beetles are the objects of numerous toxicity tests. They include not only lethality but also a broad range of effects, including altered activity of enzymes, ultrastructural malformations (Giglio et al., 2017), developmental parameters, mortality (Heimbach, 1998), and population and field effects (Huusela-Veistola, 1996; Markó and Kádár, 2005). Because of their importance in the environment as mentioned above, beetles are also used in standardized tests required for regulatory approval of veterinary and agrotechnical products, and the low toxicity of insecticides to non-target species is one of the crucial points taken under consideration. The Organization for Economic Co-operation and Development (OECD) as well as International Organization for Biological and Integrated Control (IOBC), Beneficial Arthropod Regulatory Testing Group (BART) and European and Mediterranean Plant Protection Organization (EPPO) Joint Initiative and EU developed series of tests that are used to determine the risk factors and potential adverse effects of newly introduced chemicals to non-target, beneficial arthropods. Four beetle species that are widely distributed around the globe and easy to breed in culture are used as indicators in those tests. *Aleochara bilineata* (Staphylinidae) is commonly used as a model organism for ground dwelling arthropods, *C. septempunctata* (Coccinellidae) for leaf-dwelling insects, *P. cupreus* (Carabidae) as a member of carabid beetles that are encountered frequently in agriculture sites and *Aphodius constans* (Scarabaeidae) as a model for ecologically important insects associated with fresh dung insects (Candolfi and Blümel, 2000; Römbke et al., 2007).

## Source of Bioactive Agents

### Antimicrobial Peptides

Recently, studies on compounds that protect beetles against pathogen infection have shown that this group of insects might be a useful source of antimicrobial and antitumor agents. This supposition is related to the fact that the beetles are characterized by a broad spectrum of AMPs, small bioactive compounds (usually ≤ 20 kDa) that act against bacteria, viruses, fungi, protozoa or other parasites (Vilcinskas et al., 2013; Chowański et al., 2017a). An astonishing example is the invasive harlequin ladybeetle *H. axyridis.* Vilcinskas et al. (2013) identified in this species 50 genes encoding various AMPs. However, the specificity of action very often is characteristic for the class (Chowański et al., 2017a). Regarding medical applications, the most important are defensins and their synthetic analogs (Ntwasa et al., 2012; Ishibashi, 2016). Defensins belong to the AMPs class of cysteine-rich peptides and mainly have antimicrobial activity against Gram-positive bacteria (Chowański et al., 2017a). However, some members of this AMPs class also possess activity against Gram-negative bacteria, fungi, and protozoa (Tonk et al., 2015). Their importance is associated with the fact that beetle defensins and defensin-derived AMPs exhibited promising activity against MRSA (methicillin-resistant *Staphylococcus aureus*) and *Pseudomonas aeruginosa*, bacteria that cause the largest number of nosocomial infections (Rajamuthiah et al., 2015; Ishibashi, 2016). An additional advantage of defensins compared to antibiotics is the fact that the bacteria did not develop AMPs resistance during a 30-day experiment (Iwasaki et al., 2007). Due to the above AMPs activities, clinical applications of defensin-derived AMPs have been examined, for example, as AMPs-containing bandages that suppresses MRSA proliferation (Saido-Sakanaka et al., 2005; Nakamura et al., 2011).

The potential for therapeutic application of beetle AMPs is related not only to their antimicrobial properties but also to the minimal cytotoxic effect toward normal mammalian cells. This property is associated with the fact that these compounds do not act on electrically neutral cell membranes. It should be highlighted that this specific action of defensins could be useful in anticancer treatment because several types of cancer, similar to bacteria, are characterized by negatively charged phospholipid membranes. To date, antitumor activity of defensin-derived AMPs (based on the *A. dichotoma* defensin) and harmoniasin isolated from the ladybeetle *H. axyridis* and its synthetic analog HaA4 have been confirmed, for example, in human leukemia cells (Kim et al., 2013). Their cytotoxicity is correlated with the induction of apoptosis and necrosis in the tested cell lines. CopA3, a peptide isolated from the dung beetle *Copris tripartitus*, also has similar activities. This anticancer peptide acts on human gastric cancer cells and leukemia cells (Kang et al., 2012; Lee et al., 2015).

### Non-peptide Compounds

Not only the peptides have therapeutic potential but also several non-peptide compounds. One of the best-known examples of these substances is cantharidin. Cantharidin is a terpenoid synthesized only by beetles belonging to the Meloidae (blister beetles) and Oedemeridae (pollen-feeding beetles) families (Ghoneim, 2013). Interestingly, dried body of the *Mylabris phalerata* beetle was used in traditional Chinese medicine for the treatment of many diseases (Moed et al., 2001; Yin et al., 2013). This potential medical use has been confirmed in recent studies that especially showed a broad spectrum of antitumor activity of this compound (Efferth et al., 2005; Lu et al., 2014). These results are associated with the fact that cantharidin treatment not only leads to apoptosis and necrosis of tumor cells but also causes inhibition of protein, RNA and DNA synthesis and tumor cell migration and proliferation (Sagawa et al., 2008; Hsia et al., 2016). Despite antitumor activities, cantharidin may have other biological activities, such as stimulation of bone marrow or sexual arousal (Ghoneim, 2013; Torbeck et al., 2014). However, the biggest problem with the medical usage of cantharidin is the toxicity of this compound to the mucosa, mainly the ureter, kidney and gastrointestinal tract (Yu and Zhao, 2016). However, the studies conducted by Yu and Zhao (2016) showed a very interesting perspective. In this study, the authors tested the action of cantharidin after its bioconversion by the photosynthetic bacteria *Rhodobacter sphaeroides* cultured on medium containing this compound. Bioconversion not only led to a decrease in toxicity but also increased anti activity of cantharidin against HepG-2 (human liver cancer), BEL-7406 (human liver cancer), and A549 (human lung cancer) cell lines (Yu and Zhao, 2016).

The beetle *Blaps japanensis* is also suitable as a donor of compounds that have been used for a long time for the treatment of many diseases such as fever, cough, rheumatism, cancer and inflammatory disorders. Recently, it has been revealed that the compounds responsible for those activities are called blapsols, which are active against cyclooxygenase (COX) enzymes (Yan et al., 2015). The opposite effect is provided by molossusamides extracted from *Catharsius molossus.* Research conducted by Lu et al. (2015) showed that these compounds have inhibitory properties against COX-1 and COX-2, which may be useful in anti-inflammatory therapy of chronic diseases.

The next compound that can also be considered in clinical application is pederin. Pederin is an amide with two tetrahydropyran rings detected in haemolymph and eggs of rove beetles of the genus *Paederus, Paederidus*, and *Megalopaederus* (Ghoneim, 2013). However, the beetle organism does not participate in the production of pederin. This amide is synthesized by the symbiotic bacteria *P. aeruginosa*. Like cantharidin, pederin is characterized by high toxicity, and contact of this substance with the skin leads very often to a characteristic irritation called Paederus dermatitis. Despite its toxic effects, pederin is characterized by antitumor activity. This amide, for example, induced apoptosis in human myeloid leukemia cells or HeLa cells (Samani et al., 2014; El-Hafeez and Rakha, 2017).

Other compounds that can be used in anticancer treatment are methyl-1,4-benzoquinones, ethyl-1,4-benzoquinones and 1-pentadecene isolated from the beetle *Ulomoides dermestoides*. Crespo et al. (2011) evaluated the cytotoxicity of these compounds on the A549 human lung carcinoma epithelial cell line and showed that they reduce cell viability and induce DNA damage.

Beetles are not only a source of antimicrobial and antitumor agents. Some compounds may also be useful as anticoagulants. Lee et al. (2017) described the antiplatelet and antithrombotic activity of two metabolites isolated from *T. molitor*. These compounds inhibit intrinsic blood coagulation pathways by inhibiting factor Xa (FXa), which can prevent the continuous production of thrombin and, at the same time, retain activity for primary homeostasis (Bauer, 2006).

### Other Bioactive Agents

Insect-based bioactive products can also be developed as a therapeutic agent for obesity. Seo et al. (2017) used ethanol extract of *T. molitor* larvae and revealed its anti-adipogenic and anti-obesity effect without cytotoxicity *in vitro* and *in vivo*. This extract caused up to a 90% reduction of lipid accumulation and triglyceride content in mature adipocytes of mice. Body weight gain was attenuated after oral administration of *T. molitor* larvae powder, and hepatic steatosis was reduced (Seo et al., 2017).

Xu et al. (2016) reported anticoagulant activity of crude extract of *Holotricha diomphalia* larvae (CEHDL) *in vitro* and *in vivo*. The anticoagulation activity of CEHDL was confirmed by hydrolysing fibrinogen and fibrin, for which inactivation by proteinase inhibitors, metal ions, heat and solutions with acidic or basic pH does not readily occur. CEHDL could be a promising antithrombotic agent used in medicine (Xu et al., 2016).

## Beetles as Model Organisms in Biomedicine

Insects have been used as model organisms in biomedical research for a long time. The first and most widely studied insect is undoubtedly *D. melanogaster*. Apart from Dipteran models, representatives of other orders of insects such as Lepidoptera or Orthoptera have also been used in recent years (Wilson-Sanders, 2011; Entwistle and Coote, 2018). An increasing amount of attention is devoted to the order Coleoptera, with one of the main representatives being *T. castaneum*. It has recently been used as a model in various types of biomedical research (Mukherjee et al., 2015; Schmidt-Ott and Lynch, 2016). Here, we describe the main examples.

### Neurodegenerative Diseases

Missense mutations in the human protein kinase PINK1 gene (PTEN-induced kinase 1, hPINK1) are thought to cause hereditary Parkinson’s disease with an early onset (Valente et al., 2004; Deas et al., 2009; Woodroof et al., 2011). hPINK1 differs from other protein kinases due to the presence of three unique insertions within the kinase domain and a *C*-terminal extension of an unknown function that bears no homology to any known protein domain (Kumar et al., 2017). hPINK1, which has been proposed to act as the main regulator of mitochondrial quality control and promotes the elimination of damaged mitochondria *via* autophagy (McWilliams and Muqit, 2017), is difficult to study due to its low *in vitro* kinase activity. Woodroof et al. (2011) showed that insect orthologs of PINK1 remained active *in vitro*. The PINK1 protein from the *T. castaneum* beetle (TcPINK1), despite some structural differences in comparison to hPINK1 (Woodroof et al., 2011), exhibits catalytic activity toward ubiquitin, Parkin and generic substrates (Woodroof et al., 2011; Kazlauskaite et al., 2015). It has been shown that motor defects of *Drosophila* PINK1 null flies, similar to those that occur in Parkinson’s disease, can be rescued *in vivo* by crossing lines that overexpress *Tc*PINK1 (Woodroof et al., 2011). *Tc*PINK1 is also easier to produce *in vitro* than hPINK1, which facilitates studying its structure. Study of the three-dimensional structure of the *Tc*PINK1 protein revealed details of its domain structure and function, including kinase domain loop insertions involved in controlling PINK1 activity and affecting its interactions with other proteins and the *C*-terminal domain, which is involved in the enzyme activity of PINK1. It has been shown that some of these futures are also part of the human PINK1 protein (Kumar et al., 2017). The use of *Tc*PINK1 provides molecular insights into the mechanisms of hPINK1 kinase activity and ubiquitin substrate recognition. *Tc*PINK1 was also used to investigate the impact of Parkinson’s disease-associated PINK1 missense mutations, of which nearly all are located within the kinase domain (Woodroof et al., 2011; Kumar et al., 2017). Some of these properties have been shown to be features of the human PINK1 protein (Kumar et al., 2017).

The *T. castaneum* beetle has also been used to study the function of a weakly characterized orphan cytokine receptor-like factor 3 (CRLF3). CRLF3 belongs to the family of type I cytokine receptors, which also includes the classical erythropoietin receptor (EpoR), thrombopoietin receptor, prolactin receptor or growth hormone receptor. The *Crlf3* gene is well conserved, and orthologs have been identified in vertebrates including humans and various insect species, such as *T. castaneum* and the cricket *Gryllus bimaculatus* but not *D. melanogaster* (Wyder et al., 2007; Hahn et al., 2017). Erythropoietin (Epo)-mediated neuroprotection and neuroregeneration have been described in insects: the grasshopper *Chorthippus biguttulus*, the locust *L. migratoria* (Ostrowski et al., 2011; Miljus et al., 2014), the beetle *T. castaneum* and the cricket *G. bimaculatus* but not *D. melanogaster* (Hahn et al., 2017). So far, no orthologs of Epo or the classical EpoR have been identified in invertebrate species.

Using the *T. castaneum* beetle as a simpler genetic model, it has been shown that Epo-like signaling molecules are ligands of CRLF3 and that CRLF3 plays a role in EPO-mediated neuroprotection, as knockdown of *Tc*-CRLF3 expression abolished the protective effect of Epo on *T. castaneum* brain neurons exposed to hypoxia (Hahn et al., 2017). This result suggests that the insect cytokine receptor CRLF3 serves as a neuroprotective receptor for an Epo-like cytokine and that vertebrate CRLF3, similar to its insect ortholog, may represent a tissue protection-mediating receptor. It has also been indicated that insects such as *T. castaneum* (but not *D. melanogaster*) can be used to search for additional tissue-protective Epo-receptors with conserved orthologs, as well as alternative ligands that activate neuroprotective receptors such as CRLF3 and can have mammalian orthologs with a similar function. An advantage of using insects in Epo-receptor studies is that insects do not possess erythrocytes and the classic EpoR, so studies do not have to consider interference by the abovementioned mechanisms (Hahn et al., 2017).

### Aging

Aging is a natural biological process associated with adverse changes at the molecular, cellular and tissue levels that determine the lifespans of organisms. Due to the genetic and phenotypic similarities of organisms, it is thought that the basic mechanisms of aging may be evolutionarily conserved from yeast to mammals, including humans (Barbieri et al., 2003; Bonafe et al., 2003; Bartke et al., 2013).

Insects became models of various aspects of aging and models for aging research at the tissue and genetic levels. The heart of insects, for example, has been the subject of studies related to cardiovascular diseases and aging (Ocorr et al., 2007b; Piazza and Wessells, 2011). The genetic basis of changes in heart function during aging in humans is not well-determined, partly due to the complex relationship between genes, age, disease and lifestyle (Lakatta, 2002). This complexity is the reason why simpler animal genetic models can be more useful for discovering basic mechanisms. Insects are excellent objects to study cardiac aging due to their short lifespan, many conserved genes and simple heart structure that facilitates the detection of changes. Moreover, the activity of their heart can be examined without causing premature death (Ocorr et al., 2007a) in part because oxygen is supplied through the tracheal system, which is separated from the circulatory system. Physiological changes in the endogenous activity of the insect myocardium during development and aging have been described mainly in *D. melanogaster* (Nishimura et al., 2011). It has been shown that in *D. melanogaster*, the resting heart rate declines progressively with age (Paternostro et al., 2001), the cardiac response to various stimuli is impaired in older flies (Paternostro et al., 2001; Wessells and Bodmer, 2007) and resistance to sudden stress decreases dramatically with age (Wessells et al., 2004). It has also been demonstrated that the majority of older wild-type flies exhibit non-rhythmic heart contraction patterns, including frequent asystole/bradycardia and ectopic beats (Ocorr et al., 2007b; Santalla et al., 2014). Similar changes in the heart rhythm have been detected in *T. molitor* beetles (Pacholska-Bogalska et al., 2018). In older beetles, more abnormalities in heart rhythm, arrhythmias, were detected. In this group of beetles, the incidence of arrhythmia and the arrhythmicity index were higher than in a group of younger beetles (Pacholska-Bogalska et al., 2018). This finding suggests that the beetle heart preparations can also be used as a model in the studies on aging and for testing cardioactive drugs as well.

### Host–Pathogen Interactions

The aforementioned structural and functional homologies of the immune system in insects and vertebrates, the large body size, fast development and availability of genome sequences of beetles result in the fact that beetles are very often used as models in studies concerning various aspects of host–pathogen interactions. For example, *T. castaneum* and that Gram-positive bacteria *Bacillus thuringiensis* or protozoan *Nosemia sp.* are used as a model for studying antagonistic interactions and co-evolution between hosts and their pathogens, including Red Queen-related questions (Kerstes et al., 2012; Milutinović et al., 2013). Moreover, the pair of *T. molitor* and *S. aureus* is very useful in research on immune mechanisms related to persistent infection (Dobson et al., 2012). This is a fundamental question, especially through the prism of the current problem with bacterial resistance to antibiotics.

### Pharmacology and Toxicology

Insects can be used in preclinical pharmacology research on drugs and as an early warning system to indicate epigenetic risk factors connected with the consequences caused by drugs in subsequent generations. The standard procedure to preclinical studies usually requires that at least two non-human species be tested. Searching for alternatives according to the 3 Rs – reduction, refinement and replacement of vertebrates – an increasing number of groups of researchers is looking for alternative models for research (Wilson-Sanders, 2011). Bingsohn et al. (2016) used *T. castaneum* and tested diets, which were supplemented with a psychoactive drug – valproic acid (VPA), a histone deacetylase inhibitor – on the development, longevity and reproduction of this beetle. VPA is widely used in the treatment of many human diseases such as epilepsy, migraine and bipolar disorder. It was found that in *T. castaneum*, VPA delayed development, increased female body weight and decreased fertility and fecundity in comparison to the control diet, and these results correspond to studies in vertebrate models. Even in the untreated generation of F1 beetles, the expression of epigenetic regulatory genes was induced, which confirms the potential usefulness of insects for screening for epigenetic effects of drugs (Bingsohn et al., 2016).

The pharmacological properties of cardioactive compounds can be easily tested using semi-isolated beetle hearts and the microdensitometric technique described by Gäde and Rosiński (1990). Confirmation of the usefulness of this study model and technique comes from the results obtained, for example, by Marciniak et al. (2009, 2010a), Chowański and Rosiński (2017), Chowański et al. (2017b), Lubawy et al. (2018) and others. Our group used the heart of *Z. atratus* as a non-mammalian model to detect changes caused by drugs. We used selected benzodiazepines as test compounds. Benzodiazepines are a class of drugs widely prescribed because of their pharmacological importance in relieving anxiety and insomnia and their sedative and anticonvulsant actions, which are based on allosteric modulation of the GABA receptor (Krueger, 1995; Gavish et al., 1999; Słocińska et al., 2004). The action of benzodiazepines and GABA (endogenous receptor ligand) were assayed in the semi-isolated heart of the *Z. atratus* beetle using a video microscopy technique and computer-based method of image acquisition and analysis (Marciniak and Rosiński, 2010). We observed that increasing concentrations (10^-12^ to 10^-5^ M) of benzodiazepines (Ro5-4864 and diazepam) and GABA induce chrono- and inotropic effects in the heart of *Z. atratus*. An inotropic-negative and dose-dependent action of the examined substances was observed (Słocińska et al., 2011).

The calculated effective concentration (EC_50_) is 3.9 × 10^-10^ M and 2.1 × 10^-10^ M for diazepam and GABA, respectively. However, we did not observe any significant effect of benzodiazepines on the contraction frequencies of the heart. In this case, the EC_50_ value is 2.3 × 10^-9^ M for 4′-chlorodiazepam and 1.8 × 10^-7^ M for diazepam (Słocińska et al., 2011). These data are supported by and consistent with data obtained in other animals (Surinkaew et al., 2011). Negative inotropic effects were observed with the use of benzodiazepines in various mammalian models, including papillary muscle from the right and left ventricles of guinea pigs, isolated rat and rabbit hearts, and isolated canine atrium (Holck and Osterrieder, 1985; Mestre et al., 1985; Saegusa et al., 1987; Edoute et al., 1993; Brown et al., 2008). Nevertheless, the results observed across the diverse animals confirm the insect model as a useful non-mammalian system for the evaluation changes in heart activity related to drug action or aging.

Beetles are also good models for pharmacological studies of anti-arrhythmic drugs such as digoxin. Cardiac glycosides are the featured group of plant glycosides that have strong anti-arrhythmic properties. For this reason, they are widely used as first-line treatment of various types of arrhythmias in humans and for the treatment of heart failure resulting in construction defects or abnormal heart function (Evans et al., 2009). In the case of anti-arrhythmic drugs, *Z. atratus* was found as a useful organism in research concerning cardioactivity of this group of drugs. For example, digoxin causes an anti-arrhythmic effect on *Z. atratus* myocardium. The positive chronotropic effect of digoxin increased with increasing drug concentration (10^-11^–10^-9^ M) (Kowalski, 2011). The anti-arrhythmic action of digoxin was also confirmed by the use of a pharmacological agent – terfenadine, which provokes artificial arrhythmia (Chan et al., 2009). Application of digoxin after treatment of the insect heart with terfenadine restored the basic heart rhythm.

All cardiac glycosides affect the heart, and similar effects are observed in 99% of animals and humans. To date, a similar effect of glycosides on the heart has been demonstrated in mice, rats, guinea pigs, dogs, pigs, chimpanzees and humans (Kolińska et al., 2016). The exact place of action of this class of compounds is the sodium-potassium pump (Na/K-ATPase) located in the cell membrane of cardiomyocytes. Cardiac glycosides cause an increase in energy demand of the myocardium but do not require an increase of ATP synthesis by cardiomyocytes (O’Neil, 2001). A similar manner of drug action was observed in the heart of beetles as was obtained in humans, which confirms the possibility of leading screening research on a non-mammalian model.

Several substances of plant origin are often used to modulate muscle and heart contractions (Loizzo et al., 2008). Based on the functional similarity of the insect and mammalian hearts, these substances can be first tested in insects. Several experiments were carried out on beetles to assess the cardioactive properties of glycoalkaloids extracted from plants of the *Solanaceae* family. We have shown that extract prepared from potato leaves inhibited cardiac activity in *Z. atratus* adults (Marciniak et al., 2010a). Similar activity of the extract was observed *in vivo* on pupae of the same species. The extract reduced the amplitude of heart contractions in both the retrograde and anterograde phase of myocardium activity. Moreover, changes in the duration of both phases of the heartbeat were observed (Marciniak et al., 2010a; Ventrella et al., 2015).

### Behavioral Studies

Parental care of *Nicrophorus* (Silphidae) remains a remarkable phenomenon among insects. Both parents, male and female, bury a carcass and prepare it by removing feathers and fur. Ball formed out of the carcass is next preserved with excreted anal and oral substances. After careful preparation, the female lays eggs. Hatched larvae are fed by both parents until their pupation. Despite the fact that in further larval development larvae are able to feed themselves, parents support this process to accelerate larval growth. At the same time, larvae begging is observed (Urbański, 2013). The complexity of burying beetle behavior and possibility to obtain large number of individuals provides that these beetles are one of the model organisms in the field behavioral ecology, including the physiological and molecular basis of parental care. Recently, research conducted on burying beetles clearly indicated that neurohormones and biogenic amines are crucial for the regulation of this process (Scott and Panaitof, 2004; Cunningham et al., 2015a; Panaitof et al., 2016). Moreover, a comparison between burying beetles and non-social beetles showed that the evolutionary basis of parental care is not related to the presence of specific genes but rather to methylation processes (Cunningham et al., 2015a). Burying beetles are also good model organisms in research about chemical signaling. Research conducted by Engel et al. (2016) and Steiger and Stokl (2017) proved that pheromonal signals regulated parent and offspring behavior, including inhibition of copulation and parental care synchronization.

Some coleopterans are exceptional in their social behavior. Therefore, they are objects of intensive basic studies that, in the future, may yield important information on the functioning of social animals and extrapolation of animal social behavior to human society or even in the design of robots (Miklósi and Gácsi, 2012). The first reported beetle with eusociality features was *A. incompertus* (Curculionidae) (Kent and Simpson, 1992). The biology and feeding behavior were described in a subchapter on pest management. Recent studies showed not only that one gallery is inhabited by a single core family but also that insects are divided into groups consisting of an inseminated mother, infertile female workers, larvae and their caretakers (Smith et al., 2018). Research on *Xyleborinus saxenii* confirmed that ambrosia beetles are very social. Division of labor between larvae and adults was observed. Larvae are responsible for enlarging and maintaining the hygiene the gallery; moreover, they participate in brood care (Biedermann and Taborsky, 2011).

### Other Biomedical Studies

As mentioned before, due to the homology of basic immune mechanisms, beetles are good candidates to play roles as models in a broad range of trials. This applicability also arises from the fact that in contrast to vertebrates, insects breed rapidly, produce many offspring and are more ethically acceptable, which allows them to be used for high-throughput screening, including tests concerning the effects of pharmaceuticals and other active compounds (Bingsohn et al., 2016).

The first example of such studies was a test conducted on the dung beetles *Onthophagus taurus* and *Euoniticellus fulvus*, which were fed on feces collected from sheep that were treated with ivermectin and albendazole (Wardhaugh et al., 2001). Those two substances are used as broad-spectrum antiparasitic drugs. Usage of those substances results in a decreased number of developing larvae. In insects that survived exposure to the tested drugs, delayed sexual maturation was observed. Bioassays conducted on dung beetles are especially important because they play a role in nutrient cycling and are essential to the long-term maintenance of pasture hygiene and productivity (Wardhaugh et al., 2001).

There are also many natural compounds that were in truth isolated from insects but not from beetles and possess verified activity in model beetles that can be used in further studies. One such substance is yamamarin – a pentapeptide (DILRG) isolated from the silk moth *Antheraea yamamai*. It was shown that this peptide is probably physiologically involved in the regulation of a process called diapause (Sato et al., 2010). Additionally, Walkowiak-Nowicka et al. (2018) showed the immunotropic activity of yamamarin by testing its impact on selected functions of the immune system of *T. molitor*. This compound also possesses strong cardioinhibitory effects on semi-isolated hearts of this beetle species (Szymanowska-Dziubasik et al., 2008). Thanks to formerly described activities of the pentapeptide in beetles, it was possible to show that this compound causes growth arrest in a murine leukemia cell line expressing the human gene Bcr/Abl (Sato et al., 2010) or reversible growth arrest of rat hepatoma cells due to its cytotoxic activity (Yang et al., 2007).

Another compound isolated from insect species is alloferon (HGVSGHGQHGVHG), which was found in the *Calliphora vicina* fly. According to Lee et al. (2011) and Chernysh and Kozuharova (2013), alloferon could stimulate NK cell cytotoxicity toward cancer cells, especially leukemia cells, and inhibit replication of Kaposi’s sarcoma-associated herpesvirus. When Kuczer et al. (2013, 2016) and Matusiak et al. (2014) conducted *in vivo* studies on the beetle *T. molitor*, it was found that this tridecapeptide and its structural analogs could increase PO activity and induce haemocyte apoptosis. These results indicated that beetles might be very useful model organisms for testing the cytotoxicity of various compounds.

Next, an example is the research conducted by Bourel et al. (2001) on two necrophagous beetles, *Dermestes frischi* and *Thanatophilus sinuatus*. Insects were reared on substrates containing different dosages of morphine that were calculated to create tissue concentrations similar to those encountered in human deaths due to opiate overdose. That conducted study demonstrates the potential for use of those two beetles as alternative species to use in toxicological analyses (Bourel et al., 2001).

## Summary and Perspectives

With an increasing number of fully sequenced and annotated insect genomes, omics technologies and bioinformatics can be used to exploit this huge amount of sequence information for the study of different biological aspects of insect model organisms. In the postgenomic era, the international i5K project (http://i5k.github.io/,2018) played an important role in understanding the genomes of various insect species. This project was aimed at sequencing the genome of each species known to be important in agriculture and also all known disease vectors, insects with important ecosystem functions and insects that are models in biological and biomedical research (representatives of all families).

In this review, we have provided a broad discussion of the research carried out on various beetle species and indicated the possibilities of using these insects as model organisms in the fields of molecular biology, physiology, ecophysiology, pharmacology and toxicology and indicated the importance of beetles in agriculture, food production or ecosystem monitoring (Table 1). Due to the increasing need for food for humans and harvested animals, research on that field may be an important in near future. Next, research on new generations of insecticides seem to be an intensively developing area of natural sciences.

**Table 1:** Chosen examples of beetle usage in different fields of life science.

Field of life science	Application		
Medicine	Models	Neurodegenerative disorders	Parkinson’s disease
	
		Aging	Heart aging
	
		Host–pathogen interactions	Persistent infections
	
		Pharmacology and toxicology	Benzodiazepines
	
		Behavior	Parental care
	
	Bioactive agents	Antimicrobial peptides	Defensins and their analogs
	
		Non-peptide agents	Cantharidin

Physiology	Neuroendocrinology	Study similarities between the neuroendocrine system of vertebrates and arthropods	Food intake
			Cardiovascular diseases
			Growth, development, and reproduction
			Diuresis
	
	Immunology	Study similarity of basic immune mechanisms	Phagocytosis and pathogen recognition
			Toll and Jak/STAT pathways

Agronomy	New source of nutrition	Animal feed	Aquatic feed
	
	Crop growth	Pest management, soil fertilization and reduce weeds	Toxicity of alkaloids
	
	Biomonitoring	Assessment of environmental pollution	Diversity of indicator species

The huge amount of data obtained from research conducted on beetles not only has contributed to a better understanding of the molecular and physiological mechanisms of the functioning of insect organisms but also has a significant meaning for our knowledge of the comparative aspects with regard to vertebrates. In beetles, an increasing number of new biologically active substances regulating various physiological processes have been identified, as demonstrated both in homologous and heterologous bioassays, including, to a lesser extent, in relation to vertebrate cells or tissues. We suppose that searching for counterparts of mammalian metabolic pathways and diseases in insects will rise into an intensive trend in science in the near future. For a number of new substances produced by insects, only one type of biological activity has been identified, and their wider physiological role is unknown. Because many substances often exhibit pleiotropic biological activity, there is a need to understand a wider spectrum of biological activity for newly discovered compounds. It is also necessary to develop new, specific and sensitive physiological, pharmacological or ecophysiological bioassays. In the future, broader research on beetles and their various substances will not only increase our knowledge of the functioning of insect organisms but may also have significant practical implications for biomedical applications, environmental monitoring or food production.

In the last decade, we have been observing new trends in the development of modern biotechnology, where besides medical, industrial, food and plant protection biotechnology, the term “Yellow Biotechnology” was introduced as an alternative term for insect biotechnology, opening new horizons for multidisciplinary research in the field of experimental entomology (Vilcinskas, 2013). Insect biotechnology has been defined as the use of biotechnology to develop insects, their molecules, cells or organs into products and services for specific applications in medicine, plant protection and industry. Beetles (e.g., *Tribolium confusum, T. castaneum, T. molitor*, and other beetles) that feed on crops or stored products are still the most important competitors for human nutrition on a global scale. Therefore, for practical reasons, there is a need for further development of new bioassays using model beetle species for not only those feeding on plants and stored products but also those species with potentially high biomedical or industrial significance.

## Author Contributions

All the authors are responsible for the general idea of the manuscript and text editing. ZA and SC are responsible for the formal edition of the manuscript. MSł coordinated the introduction. MSz, JP-B, PM, KW-N, AU, and MSł coordinated a description of neuroendocrinological, immunological, pharmacological aspects, aging, model organisms, and bioactive agents. MSp, SC, JL, and ZA coordinated the description of beetles in agronomy and ecology. GR coordinated the summary and perspectives.

## Conflict of Interest Statement

The authors declare that the research was conducted in the absence of any commercial or financial relationships that could be construed as a potential conflict of interest.
